# Using random forest algorithm for glomerular and tubular injury diagnosis

**DOI:** 10.3389/fmed.2022.911737

**Published:** 2022-07-28

**Authors:** Wenzhu Song, Xiaoshuang Zhou, Qi Duan, Qian Wang, Yaheng Li, Aizhong Li, Wenjing Zhou, Lin Sun, Lixia Qiu, Rongshan Li, Yafeng Li

**Affiliations:** ^1^School of Public Health, Shanxi Medical University, Taiyuan, China; ^2^Department of Nephrology, Shanxi Provincial People’s Hospital (Fifth Hospital) of Shanxi Medical University, Taiyuan, China; ^3^Shanxi Provincial Key Laboratory of Kidney Disease, Taiyuan, China; ^4^School of Medical Sciences, Shanxi University of Chinese Medicine, Jinzhong, China; ^5^College of Traditional Chinese Medicine and Food Engineering, Shanxi University of Chinese Medicine, Jinzhong, China; ^6^Core Laboratory, Shanxi Provincial People’s Hospital (Fifth Hospital) of Shanxi Medical University, Taiyuan, China; ^7^Academy of Microbial Ecology, Shanxi Medical University, Taiyuan, China

**Keywords:** random forest, machine learning, auxiliary diagnosis, glomerular injury, tubular injury

## Abstract

**Objectives:**

Chronic kidney disease (CKD) is a common chronic condition with high incidence and insidious onset. Glomerular injury (GI) and tubular injury (TI) represent early manifestations of CKD and could indicate the risk of its development. In this study, we aimed to classify GI and TI using three machine learning algorithms to promote their early diagnosis and slow the progression of CKD.

**Methods:**

Demographic information, physical examination, blood, and morning urine samples were first collected from 13,550 subjects in 10 counties in Shanxi province for classification of GI and TI. Besides, LASSO regression was employed for feature selection of explanatory variables, and the SMOTE (synthetic minority over-sampling technique) algorithm was used to balance target datasets, i.e., GI and TI. Afterward, Random Forest (RF), Naive Bayes (NB), and logistic regression (LR) were constructed to achieve classification of GI and TI, respectively.

**Results:**

A total of 12,330 participants enrolled in this study, with 20 explanatory variables. The number of patients with GI, and TI were 1,587 (12.8%) and 1,456 (11.8%), respectively. After feature selection by LASSO, 14 and 15 explanatory variables remained in these two datasets. Besides, after SMOTE, the number of patients and normal ones were 6,165, 6,165 for GI, and 6,165, 6,164 for TI, respectively. RF outperformed NB and LR in terms of accuracy (78.14, 80.49%), sensitivity (82.00, 84.60%), specificity (74.29, 76.09%), and AUC (0.868, 0.885) for both GI and TI; the four variables contributing most to the classification of GI and TI represented SBP, DBP, sex, age and age, SBP, FPG, and GHb, respectively.

**Conclusion:**

RF boasts good performance in classifying GI and TI, which allows for early auxiliary diagnosis of GI and TI, thus facilitating to help alleviate the progression of CKD, and enjoying great prospects in clinical practice.

## Introduction

Chronic kidney disease (CKD) is a common chronic condition worldwide, with a prevalence of 13.4% (1). Due to its imperceptible symptoms at the initial stages, it may progress into end-stage renal disease, which requires kidney transplantation, posing a substantial financial burden to the society leading to a lower quality of life and higher mortality rate. Additionally, it’s highly associated with such complications as cardiovascular disease (2), emerging as another “silent killer” that threatens human life after tumors and diabetes. Renal injury is the prerequisite for CKD, including glomerular injury (GI) and tubular injury (TI), and thus early diagnosis of GI and TI is of practical significance to alleviate the progression of CKD. How to better make an early diagnosis of renal injury is now a topic running into the forefront of research.

In 2012, Luxia Zhang employed logistic regression to predict CKD, but it comes with drawbacks (3). The first one concerns its sensitivity to multicollinearity; the second one is that maximum likelihood estimation does not fit the true distribution of the data well. A better model is needed. With artificial intelligence springing up, data-driven algorithms pick up pace, and have become a research hotspot in the life sciences, enjoying great popularity in cardiovascular diseases (4), tumors (5), immune diseases (6), and neurological diseases (7). Also, its application in renal diseases is on the rise, from acute kidney injury prediction (8) to kidney transplantation outcome prediction (9), interstitial fibrosis, and tubular atrophy detection (10). One of the well-known algorithms represents Random forest (RF), which has been shown a powerful tool in disease auxiliary diagnosis (11, 12). However, it has not yet been determined in glomerular and tubular injury.

Feature selection is of great necessity in constructing classifiers, since the presence of irrelevant features may be responsible for the poor model performance (13). L1 regularization, Absolute Shrinkage and Selection Operator (LASSO) regression is a welcome choice. It is characterized by the inclusion of an L1 regularization penalty term in fitting generalized linear regression, which makes the sum of the absolute values of the regression coefficients of the model lower than a particular value. It aims to minimize the sum of squared residuals, forcing the regression coefficients of variables that contribute less to the model to be compressed to zero and achieving a feature sparse process (14, 15). Also, it could eliminate predictors with autocorrelation or redundancy, allowing for automated variable selection within the model, and significantly contributing to the performance of classification models (16, 17). Another headwind in the development of classifiers relates to imbalanced datasets. It is not unusual in medical research, because the number of non-patients is extremely larger than that of patients, which serves as an obstacle to predictive performance (18). RF is sensitive to response variables with unbalanced data, and imbalances in classes in the data tend to tend to larger classes in the output of the model, resulting in some classification errors, resulting in lower classification accuracy (19). It has been documented that machine learning methods with data balancing techniques represent effective approach for stroke prediction with imbalanced data. As such, it’s crucial to balance the data prior to model construction (20).

In this study, we aimed to (1) employ LASSO algorithm to conduct feature selection for GI and TI; (2) use the classical and widely accepted SMOTE (Synthetic Minority Over-sampling Technique) algorithm to handle the imbalanced classes of GI and TI; (3) employ the mature machine learning algorithm, Random Forest (RF) to make a classification of GI and TI, respectively, and compare its performance with logistic regression (LR) and Naive Bayes (NB), thus achieving the auxiliary diagnosis of GI and TI and providing a new idea for clinical practice in delaying the progression of CKD.

## Participants and methods

### Study participants

Shanxi Provincial People’s Hospital conducted CKD screening for permanent residents aged ≥ 40 years in the northern region of Shanxi Province (Ningwu County), the central regions (Yu County, Yangqu County, Lin County, Shouyang County), and the southern regions (Zezhou County, Huozhou City, Hejin City, Linyi County, and Ruicheng County) from April 2019 to November 2019. A total of 13,550 residents volunteered for this screening, and 12,285 were eventually enrolled in the study, including 5,206 men and 7,079 women aged 41–91 years.

Inclusion criteria: (1) residents aged 40 years or older; (2) conscious participants without communication impairment; (3) participants understanding the significance of the study and willing to sign a written informed consent; (4) participants with no cognitive impairment or mental illness; (5) more than 1 year of local residents as of the survey date. Exclusion criteria: (1) Severely incomplete information recorded; (2) poor compliance; (3) pregnant women or those with a history of substance abuse.

### Data collection

Questionnaires, physical examination, and laboratory analysis were used to collect data. (1) The questionnaire comprised demographic information (including age, sex, annual income, educational levels), lifestyle (including smoking, alcohol consumption, diet, and exercise). The questionnaire was administered online and completed by the subjects themselves or their family members. (2) Physical examination included height, weight and blood pressure (systolic blood pressure, diastolic blood pressure), which were measured twice and then the mean value was calculated. All data were measured by a medical professional. Body mass index (BMI) was calculated by weight in kilograms divided by the square of height in meters.

(3) Fasting venous blood was collected from subjects for fasting blood glucose (FPG), glycated hemoglobin (GHb), homocysteine (Hcy), total cholesterol (TC), triglycerides (TG), low-density lipoprotein cholesterol (LDL-C) and high-density lipoprotein (HDL-C). (4) Morning urine specimens were collected from subjects. After centrifugation at 3,000 r/min for 10 min, the supernatant was extracted (low-speed centrifuge Anhui Zhongke Zhongjia SC3616), and α1-microglobulin (α1MG), urinary creatinine (UCr), and microalbuminuria (MAU) were determined by latex turbidimetry, sarcosine oxidase, and immunoturbidimetry, respectively.

### Variable assignments

Information on the annual income, educational levels, health history, and lifestyle of the study participants was obtained from the questionnaire. Annual income was defined as < 5K yuan, 5K–10K yuan, 10K–20K yuan, > 20K yuan; education levels were defined as ≤ primary school, ≤ middle school, ≤ high school, ≥ bachelor’s degree; smoking was classified as yes or no; alcohol consumption was classified as always (>100 g/time and 3 times/week), sometimes (<3 times/week or < 100 g/time) and rarely; exercise was classified as “none or a little” or “regular” (≥3 times/week, ≥ 30 min/time). BMI was defined as underweight (<18.5 kg/m^2^), normal weight (18.5–24.0 kg/m^2^), overweight (24.0–28.0 kg/m^2^), obesity (≥28 kg/m^2^). ACR was defined as urinary microalbumin divided by urinary creatinine multiplied 8.84; MCR was defined as urinary microglobulin divided by urinary creatinine multiplied 8.84.

#### Explanatory variables

(1) Questionnaire: demographic information (age, sex, educational levels, annual income, residence, etc.); lifestyle (smoking, alcohol, exercise, salt consumption, diet). (2) Morning blood: HDL, LDL, TG, TC, Hcy, FPG, GHb. (3) Physical examination: SBP, DBP, BMI. 20 variables in total.

#### Response variables

ACR ≥ 30 mg/g was defined as GI; MCR > 23 mg/g was defined as TI. The presence of GI, TI was assigned 1; otherwise, they were defined as 0. In this study, we employed RF, LR and NB to make a classification of GI and TI, respectively.

### L1 regularization, absolute shrinkage and selection operator regression

Absolute Shrinkage and Selection Operator (LASSO) is one of the common methods for feature selection. It is characterized by the inclusion of an L1 regularization penalty term in fitting generalized linear regression, which makes the sum of the absolute values of the regression coefficients of the model lower than a particular value. It aims to minimize the sum of squared residuals, forcing the regression coefficients of variables that contribute less to the model to be compressed to zero and achieving a feature sparse process (14, 15). LASSO was used to select the collected explanatory variables, and to determine those more relevant to the response variables.

### Synthetic minority over-sampling technique algorithm

**FIGURE 1:**
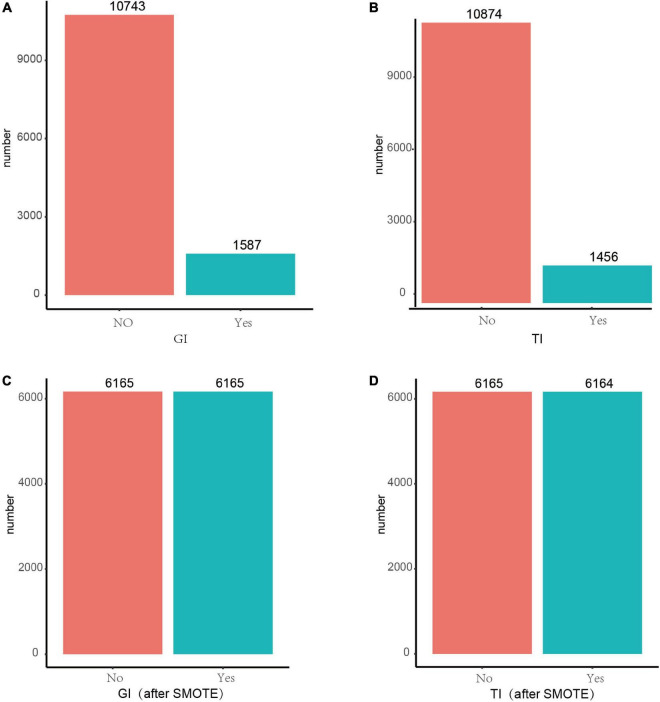
Before and after SMOTE of response variables for GI and TI. SMOTE, Synthetic Minority Over-Sampling Technique. It’s a good and powerful way to handle imbalanced data, and it was conducted under the parameters of *k* = 5, C.perc = “balance,” dist = “Overlap.” **(A)** GI before SMOTE; **(B)** TI before SMOTE; **(C)** GI after SMOTE; **(D)** TI after SMOTE.

The Synthetic Minority Over-Sampling Technique (SMOTE) is an oversampling technique that is an effective algorithm for dealing with imbalances between data classes (21). It’s employed to synthetically enlarge the minority class using K-nearest neighors to obtain a balanced data set (22) and has been shown good performance in such fields as network intrusion detection systems and disease detection. In this study, there is a serious imbalance in the response variables, GI and TI (Figures 1A,B). SMOTE was used to balance the classes to facilitate the machine learning models to better learn the inter-data features, thus making the best classification judgment.

### Random forest

**FIGURE 2:**
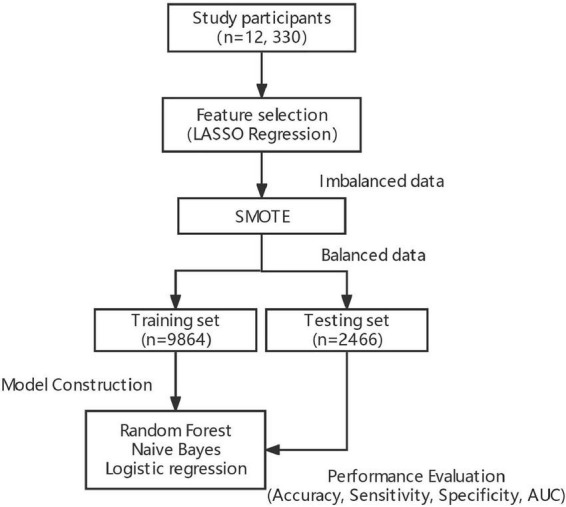
Workflow of the model construction.

RF, a data-driven integrated learning algorithm, could obtain multiple new training data by an autonomous sampling of the training set, constructing multiple classification trees based on the parallelization of these new data, and achieving de-correlation between the trees by introducing the selection of independent variables. By doing so, the diversity of the classification trees originates from both sample and independent variable perturbations to achieve the effect of reducing the model variance, and finally to vote on the classification results of multiple trees to obtain the final classification results (23, 24). The workflow of the model construction is shown in Figure 2.

### Statistical methods

#### Statistical description

Qualitative data are expressed as percentages (%), and quantitative data are expressed as mean ± standard deviation (M±*SD*) or median ± interquartile [(*Median*(*P25*,*P75*))], as appropriate.

#### Model construction

The datasets were divided into training set (80%) and testing set (20%). The former ones were used for models training, i.e., RF, NB, and LR, while the latter ones were employed for evaluation of model performance. All analyses were implemented in R software (version 4.0.3).

### Evaluation parameters

The evaluation parameters comprised Accuracy (1), Specificity (2), Sensitivity (3) and area under the receiver operating curve (AUC). The predicted result was defined as True Positive (TP) when patients with renal conditions were classified as patients and True Negative (TN) when healthy ones were classified as healthy. Besides, the predicted result was False Positive (FP) if healthy subjects are considered patients; similarly, False Negative (FN) if patients are considered healthy subjects. Accuracy is to evaluate how accurate the machine learning algorithms are to detect what it is supposed to measure. Specificity is the ability to correctly exclude those without renal conditions and Sensitivity is to correctly identify those with renal conditions.


$$\begin{array}{c} Accuracy=\frac{\left(TN+TP\right)}{\left(TP+TN+FP+FN\right)}\times 100\%\left(1\right) \end{array}$$

$$\begin{array}{c} Specificity=\frac{TN}{\left(TN+FP\right)}\times 100\%\left(2\right) \end{array}$$

$$\begin{array}{c} Sensitivity=\frac{TP}{\left(TP+FN\right)}\times 100\%\left(3\right) \end{array}$$

## Results

### Baseline characteristics

A total of 12,330 people were included in this study, 5,230 men and 7,100 women, the number of GI was 1,587 (12.8%) and the number of TI was 1,456 (11.8%). Besides, the number of participants with both GI and TI was 2,439 (19.7%). Other parameters are detailed in Tables 1, 2.

**TABLE 1:** Clinical parameters of study subjects (quantitative ones).

Variables	$\bar{x}$ ± *s*	Variables	$\bar{x}$ ± *s*
Age(y)	58.75 ± 9.49	TC (mmol/L)	4.43 ± 0.95
LDL (mmol/L)	2.35 ± 0.84	TG (mmol/L)	1.73 ± 0.82
HDL (mmol/L)	1.30 ± 0.37	Hcy (mmol/L)	22.98 ± 14.26
FPG (mmol/L)	4.97 ± 1.36	SBP (mmHg)	136.10 ± 18.39
GHb (mmol/L)	5.54 ± 1.09	DBP (mmHg)	82.84 ± 10.76

**TABLE 2:** Clinical parameters of study subjects (qualitative ones).

Variables	Percentage (%)	Variables	Percentage (%)	Variables	Percentage (%)
**Education**		**BMI**		**Income (Yuan)**	
≤Primary	32.7	Underweight	1.7	<5k	41.8
≤Junior	50.9	Normal	39.5	5k–10k	25.5
≤Senior	11.9	Overweight	42.6	10k–20k	10.3
≥Bachelor	4.5	Obesity	16.3	>20k	22.4
**Salt consumption**		**Alcohol**		**Diet**	
Light	26.3	Rarely	84.7	Vegetable	33.5
Moderate	60.5	Sometimes	13.2	Balanced	61.9
Salty	13.1	Always	2.1	Meat	4.6
**Exercise**		**Smoking**		**Sex**	
Regular	41.7	No	76.2	Male	42.4
None or a little	58.3	Yes	23.8	Female	57.6

### Feature selection and results of sampling technique

**FIGURE 3:**
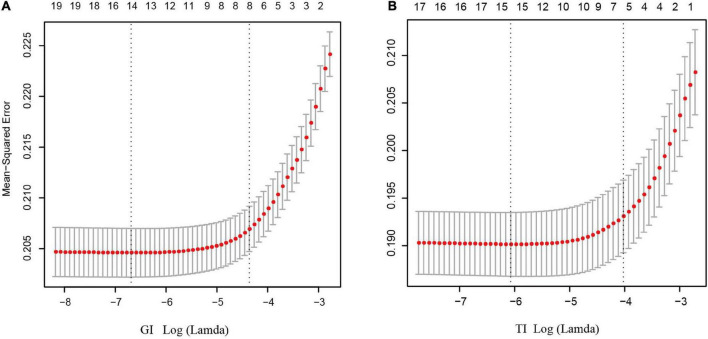
Results of feature selection using LASSO. When Lamda is minimum, corresponding features were taken into model construction (14 features for GI, and 15 feature for TI). **(A)** Feature selection for GI; **(B)** feature selection for TI.

As shown in Figures 3A,B, after LASSO feature selection, 14 and 15 explanatory variables remained in the two datasets, respectively, in which data 1 with GI as the response variable excluded six variables of annual income, residence, LDL, HDL, smoking, and exercise; while data 2 with TI as the response variable excluded five variables of TC, LDL, HDL, exercise, and salt consumption. The remained variables are comparable for GI (except TG and sex) and TI (Supplementary Tables 1, 2).

As shown in Figures 1C,D, after resampling by SMOTE, the number of patients and normal ones were 6,165, 6,165 for GI, and 6,165, 6,164 for TI, espectively.

### Model performance

**FIGURE 4:**
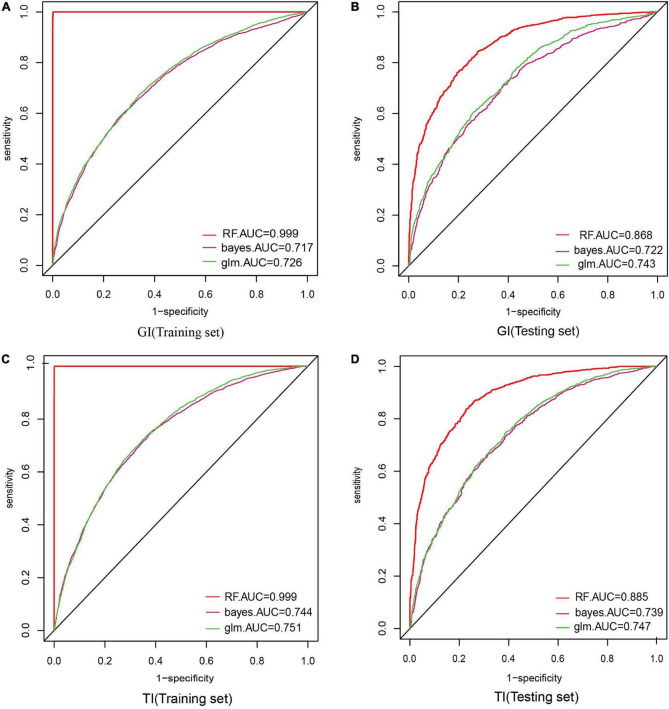
Comparison of the ROC curve areas of the three model classifiers. In model construction, 70% of samples were randomly divided as training set, and the rest 30% were as testing set. AUC (area under curve) was used to evaluate the performance of these three classifiers. **(A)** AUC of GI in the training set; **(B)** AUC of GI in the testing set; **(C)** AUC of TI in the training set; **(D)** AUC of TI in the testing set.

When constructing model for GI, the number of GI and non-GI in the training set were both 4,932, and 1,233 in the testing set, respectively. When constructing model for GI, the number of TI and non-TI in the training set were 4,973 and 4,891, respectively, and 1,273 and 1,192 in the testing set. The accuracy, sensitivity, specificity and AUC of RF for classification of GI and TI performed better than NB and LR in both the training set and testing set, which shows that RF does have a high diagnostic value for classification (Tables 3, 4 and Figure 4).

**TABLE 3:** Performance evaluation of the three classifiers on the training set (GI/TI).

Model	Accuracy (%)	Sensitivity (%)	Specificity (%)
RF	99.90/99.92	99.96/99.94	99.84/99.90
NB	65.39/67.06	52.08/54.26	78.71/79.65
LR	66.40/68.52	64.90/66.94	67.90/70.08

**TABLE 4:** Performance evaluation of the three classifiers on the testing set (GI/TI).

Model	Accuracy(%)	Sensitivity(%)	Specificity(%)
RF	78.14/80.49	82.00/84.60	74.29/76.09
NB	65.17/65.68	52.23/53.34	78.10/78.86
LR	66.87/67.51	64.23/66.06	69.51/69.04

### Feature importances

**FIGURE 5:**
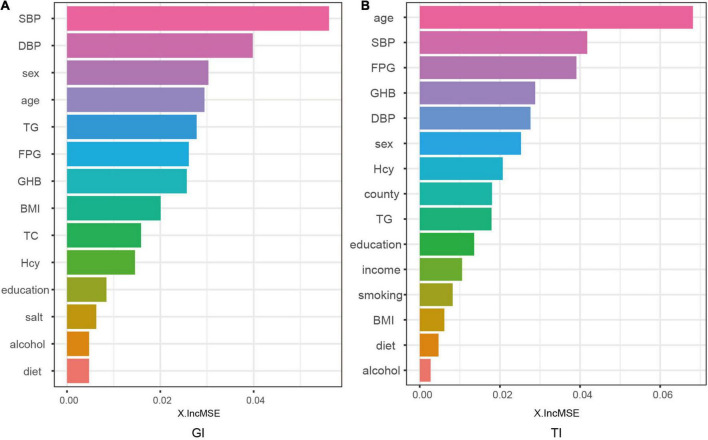
Contributions of explanatory variables to the random forest model. The “%IncMSE” is the increase in mean squared error, where the error of the model prediction is increased by randomly replacing the value of each predictor variable if it is more important. Therefore, a larger value indicates that the variable is more important.

We indicated the contribution of the explanatory variables to the model by %IncMSE, and the larger the %IncMSE, the more important the variables were for the RF model. The four variables that contributed most to the classification of GI in the RF model represented SBP, DBP, sex, and age. The four variables that were most important for the classification of TI constituted age, SBP, FPG, and GHb (Figure 5).

## Discussion

In this study, ACR and MCR levels were used as screening indicators for GI and TI, and ACR ≥ 30 mg/g was considered GI; MCR > 23 mg/g was considered TI. Besides, the machine learning model RF was used to classify them, and we compared its classification performance with NB, and LR.

This study suggested that the accuracy, sensitivity, specificity and AUC of the RF algorithm outperformed other classifiers in both the training set and testing set. Yet its performance in the testing set was comparatively lower than that in the training set, because the classification performance based on the training set was prone to overfitting (25), while the results of the testing set could better reflect the classification performance of the model, which proves their potential applications in GI and TI-aided diagnosis.

Of note, the RF model is sensitive to response variables with unbalanced data. Imbalanced classes in the data would leave the output of the model tending to larger classes, causing some classification errors, and leading to a less accurate classification performance (20). As such, SMOTE algorithm was employed to resample the data set with GI and TI as response variables, respectively, before performing the classification task to achieve balanced classes. By doing so, the learning capability of the model could be maximized, and a more accurate predictive performance could be achieved.

Since RF model is data-driven, a visible functional equation is unavailable to determine the extent to which the explanatory variables contribute to the model based on the regression coefficients. However, a more intuitive alternative for the model is that by outputting feature importances, the model could explain the importance of the variables on the explanatory variables. This study demonstrated that the four explanatory variables with the greatest output weight of RF classifier for GI represented SBP, DBP, sex, and age; and the four explanatory variables for TI constituted age, SBP, FPG, and GHb.

In a hypertensive state, abnormal glomerular hemodynamics, spasmodic constriction of renal arteries would reduce renal blood flow, leading to renal ischemia and long-term hyperperfusion and hyperfiltration of glomerular capillaries, resulting in damage to glomerular vascular endothelial cells and podocytes (26, 27). Meanwhile, renal ischemia caused by hypertension activates the renin-angiotensin-aldosterone system, leading to constriction of the inlet and outlet arteries and a further increase in glomerular pressure, which aggravates renal ischemia. The high pressure causes damage to endothelial cells, podocytes and tubular epithelial cells, leading to the destruction of the filtration barrier and dysfunction of reabsorption, thus, resulting in proteinuria occurrence (28, 29). Additionally, hypertension can lead to thickening of glomerular duct wall hardening, renal parenchymal ischemia, which would further increase the production of vasoactive substances, stimulate interstitial collagen deposition, and eventually leading to glomerular sclerosis and kidney injury (30).

It has been documented that kidney disease in China is more prevalent in male patients, suggesting that sex is also one contributor to degenerative changes in kidney structure and function. The prevalence of CKD has been reported to be higher in women than in men. A representative study pooling 33 population-based studies worldwide evaluated the global prevalence of stage 1–5 CKD at 10.4% among men and 11.8% among women aged 20 years and older (31). The reasons for these differences are unclear, and although the GFR estimation equation includes a gender correction factor, a single threshold value of < 60 ml/min per 1.73 m^2^ for the definition of CKD may lead to overdiagnosis of CKD in women (32). A follow-up of Swedish patients with CKD not on dialysis in the national registry showed that male patients had a faster decline in eGFR, more rapid CKD progression and higher all-cause mortality compared to women (33). Also. the results of a study are consistent with experimental data showing the protective effect of estrogen and the potentially deleterious effect of testosterone on non-diabetic CKD (34). The effect of gender on CKD incidence, prevalence, and progression needs further study, and the development of gender-specific CKD markers is also a hot topic of current research.

After the age of 40, the glomerular filtration rate decreases at a rate of 1 ml/min/1.73 per year, resulting in stiffening of the renal vessel wall, glomerular atrophy, sclerosis, tubular atrophy, and interstitial fibrosis, which eventually lead to renal hypofunction (35). In diabetic patients, high blood glucose concentration would cause glucose metabolism disorder, hemodynamic changes, oxidative stress, which induce renal tubular epithelial cell hypertrophy, tubular basement membrane destruction, interstitial cell infiltration, and renal tubular interstitial fibrosis, contributing to reabsorption dysfunction (36, 37). Therefore, the present study shows that RF has some clinical practice combined with feature selection by LASSO.

There are also some limitations in this paper. Firstly, the study constructed the models with data from Shanxi Province, and no other external datasets are available to validate the model performance. Our ongoing work is to collect samples from other areas, to validate the generalization capabilities of the model. Secondly, this study was initially considered for cost-effectiveness and other indicators reflecting CKD were not collected, such as blood creatinine, which will also be the focus of our next step. Additionally, as CKD was more prevalent in people aged ≥ 40 years, this study centered on those over 40 years. In the future, we consider surveys on those aged 18–40 years to improve the prediction model in younger groups. Finally, GI and TI were defined only by surrogate parameters, ACR and MCR, which may not well accurately reflect the renal conditions. In our future work, we would conduct a follow-up for those with positive urine protein.

In short, as early manifestations of CKD, GI and TI have emerged as a global public health issue; their early diagnosis and corresponding treatment are of great importance. Our results demonstrate the potential value of machine learning algorithms in GI and TI-assisted diagnosis, which facilitates reducing the workload of doctors, while achieving automated diagnosis and treatment decisions, and thus could be promoted in clinical practice.

## Data availability statement

The raw data supporting the conclusions of this article will be made available by the authors, without undue reservation.

## Ethics statement

The studies involving human participants were reviewed and approved by the Ethics Committee of Shanxi Provincial People’s Hospital. The patients/participants provided their written informed consent to participate in this study.

## Author contributions

WS was responsible for the data analysis and the writing of the manuscript. XZ, QD, QW, and YahL helped polish the manuscript. AL, WZ, LS, LQ, and RL gave precious advice on the statistical methods. YafL was responsible for the conception and design of the research. All authors read and approved the final draft.
