# Supplementary material for: Using random forest algorithm for glomerular and tubular injury diagnosis
Source: Front Med (Lausanne). 2022 Jul 28;9:911737. doi: 10.3389/fmed.2022.911737 (PMC9366016; doi:10.3389/fmed.2022.911737)
Supplement: Supplementary file 1 [file Data_Sheet_1.docx]

Supplementary Table 1 Comparisons of quantitative clinical indexes between training set and testing set

|  | GI | | | TI | | |
| --- | --- | --- | --- | --- | --- | --- |
|  | Training(N=9864) | Testing(N=2466) | *P* | Training(N=9864) | Testing(N=2466) | *P* |
| Age(y) | 59.00(52.00,67.00 ) | 59.00(53.00, 67.00) | 0.199 | 61.00(54.00, 68.00) | 60.00(54.00, 68.00) | 0.147 |
| TG(mmol/L) | 1.63(1.17, 2.27) | 1.69(1.20, 2.34) | 0.02 | 1.61(1.15, 2.23) | 1.66(1.15, 2.34) | 0.052 |
| TC(mmol/L) | 4.42(3.78, 5.08) | 4.39(3.77, 5.04) | 0.273 |  |  |  |
| FPG(mmol/L) | 4.80(4.30, 5.50) | 4.80(4.30, 5.60) | 0.333 | 4.80(4.30, 5.50) | 4.80(4.30, 5.50) | 0.611 |
| GHB(mmol/L) | 5.40(5.00, 5.90) | 5.40(5.00, 6.00) | 0.17 | 5.40(5.00, 6.00) | 5.40(5.10, 6.00) | 0.749 |
| SBP(mmHg) | 137.50(126.00, 152.00) | 137.50(125.00, 152.00) | 0.948 | 137.00(126.00, 151.00) | 137.00(125.00, 151.00) | 0.813 |
| DBP(mmHg) | 83.00(77.50, 91.00) | 83.000(77.50, 91.00) | 0.529 | 82.00(77.00, 90.00) | 82.50(77.50, 90.50) | 0.113 |
| Hcy(mmol/L) | 18.60(13.60,27.90 ) | 18.55(13.60, 28.13) | 0.611 | 19.30(14.00, 29.60) | 19.10(13.80, 29.05) | 0.087 |

Supplementary Table 2 Comparisons of qualitative clinical indexes between training set and testing set

| Variables | GI | | | TI | | |
| --- | --- | --- | --- | --- | --- | --- |
|  | Training(N=9864) | Testing(N=2466) | *P* | Training(N=9864) | Testing(N=2466) | *P* |
| **Education** |  |  |  |  |  |  |
| ≤primary | 3591(36.4) | 860(34.9) | 0.093 | 3749(38.0) | 924(37.5) | 0.554 |
| ≤junior | 4929(50.0) | 1288(52.2) |  | 4829(49.0) | 1193(48.4) |  |
| ≤senior | 969(9.8) | 243(9.9) |  | 974(9.9) | 26110.6 () |  |
| ≥bachelor | 375(3.8) | 75(3.0) |  | 312(3.2) | 87(3.5) |  |
| **Exercise** |  |  |  |  |  |  |
| regular | 4215(42.7) | 1026(41.6) | 0.312 |  |  |  |
| none or a little | 5649(57.3) | 1440(58.4) |  |  |  |  |
| **BMI** |  |  |  |  |  |  |
| underweight | 158(1.6) | 34(1.4) | 0.784 | 165(1.7) | 41(1.7) | 0.748 |
| normal | 3426(34.7) | 867(35.2) |  | 3753(38.0) | 951(38.6) |  |
| overweight | 4225(42.8) | 1041(42.2) |  | 4220(42.8) | 1026(41.6) |  |
| obesity | 2055(20.8) | 524(21.2) |  | 1726(17.5) | 447(18.1) |  |
| **Alcohol** |  |  |  |  |  |  |
| Rarely | 8677(88.0) | 2129(86.3) | 0.05 | 8329(84.4) | 2095(85.0) | 0.09 |
| Sometimes | 1009(10.2) | 294(11.9) |  | 1327(13.5) | 304(12.3) |  |
| Always | 178(1.8) | 43(1.7) |  | 208(2.1) | 66(2.7) |  |
| **Smoking** |  |  |  |  |  |  |
| No | 7849(79.6) | 1943(78.8) | 0.391 | 7267(73.7) | 1824(74.0) | 0.744 |
| Yes | 2015(20.4) | 523(21.2) |  | 2597(26.3) | 641(26.0) |  |
| **Diet** |  |  |  |  |  |  |
| Vegetable | 3273(33.2) | 831(33.7) | 0.798 | 3448(35.0) | 835(33.9) | 0.569 |
| Balanced | 6188(62.7) | 1530(62.0) |  | 6024(61.1) | 1534(62.2) |  |
| Meat | 403(4.1) | 105(4.3) |  | 392(4.0) | 96(3.9) |  |
| **Salt consumption** |  |  |  |  |  |  |
| Light | 2606(26.4) | 678(27.5) | 0.173 |  |  |  |
| Moderate | 6065(61.5) | 1467(59.5) |  |  |  |  |
| Salty | 1193(12.1) | 321(13.0) |  |  |  |  |
| **Sex** |  |  |  |  |  |  |
| Male | 3523(35.7) | 934(37.9) | 0.046 | 4551(46.1) | 1124(45.6) | 0.631 |
| Female | 6341(64.3) | 1532(62.1) |  | 5313(53.9) | 1341(54.4) |  |
